# Perioperative Intravenous Amino Acid Infusion in Major Urologic Surgery

**DOI:** 10.3390/jcm12206614

**Published:** 2023-10-19

**Authors:** Claudia Brusasco, Fabio Maria Valenzi, Marco Micali, Marco Ennas, Antonia Di Domenico, Federico Germinale, Federico Dotta, Andrea Benelli, Fabio Campodonico, Giada Cucciolini, Antonio Carbone, Carlo Introini, Francesco Corradi

**Affiliations:** 1 Anaesthesia and Intensive Care Unit, E.O. Ospedali Galliera, 16128 Genoa, Italy; marco.micali@galliera.it; 2 Urology Unit, Department of Medico-Surgical Sciences & Biotechnologies, Faculty of Pharmacy & Medicine, Sapienza University of Rome, 04100 Latina, Italy; fabiovalenzi@gmail.com (F.M.V.); antonio.carbone@uniroma1.it (A.C.); 3 Urology Unit, E.O. Ospedali Galliera, 16128 Genoa, Italy; marco.ennas@galliera.it (M.E.); antonia.didomenico@galliera.it (A.D.D.); federico.germinale@galliera.it (F.G.); federico.dotta@galliera.it (F.D.); andrea.benelli@galliera.it (A.B.); fabio.campodonico@galliera.it (F.C.); carlo.introini@galliera.it (C.I.); 4 Department of Surgical, Medical, Molecular Pathology and Critical Care Medicine, University of Pisa, 56126 Pisa, Italy; giada.cucciolini@phd.unipi.it (G.C.); francesco.corradi@unipi.it (F.C.)

**Keywords:** amino acid, post-operative acute kidney injury, post-operative complications, ERAS programs, ERAS protocols, enhanced recovery after surgery, urologic surgery, minimally invasive surgery

## Abstract

Post-operative acute kidney injury (PO-AKI) is a serious complication that may occur after major abdominal surgery. The administration of intravenous perioperative amino acids (AAs) has been proven to increase kidney function and has some beneficial effects to prevent PO-AKI. The aim of this study was to establish if the perioperative infusion of AAs may reduce the incidence of PO-AKI in patients undergoing major urological minimally invasive surgery. From a total of 331 patients, the first 169 received perioperative crystalloid fluids and the following 162 received perioperative AA infusions. PO-AKIs were much higher in the crystalloid group compared to the AA group (34 vs. 17, *p* = 0.022) due to a lower incidence of KDIGO I and II in the AA group (14 vs. 30 *p* = 0.016). The AA group patients who developed a PO-AKI presented more risk factors compared to those who did not (2 (2-4) vs. 1 (1-2), *p* = 0.031) with a cut-off of 3 risk factors in the ROC curve (*p* = 0.007, sensitivity 47%, specificity 83%). The hospital length of stay was higher in the crystalloid group (*p* < 0.05) with a consequent saving in hospital costs. Perioperative AA infusion may help reduce the incidence of PO-AKI after major urological minimally invasive surgery.

## 1. Introduction

Post-operative acute kidney injury (PO-AKI) is defined as a decrease in kidney function occurring within seven days from surgery and is one of the most frequent post-operative complications after major abdominal surgery. In major laparoscopic urologic surgery, PO-AKIs have been described to occur in 15.5% of cases [1]. PO-AKIs, diagnosed according to the KDIGO classification [2,3], are associated with an increased risk of short-term adverse outcomes, including dialysis, cardiovascular events, lung injury, delirium and infection; all these adverse events can lead to increased long-term morbidity and mortality [4]. PO-AKIs commonly have a multifactorial etiology with pre-operative, intra-operative and post-operative factors increasing PO-AKI risk. The most important pathophysiologic mechanisms responsible for PO-AKIs involve alterations in kidney microcirculation, an increased oxygen demand, hypoperfusion and systemic inflammatory reactions to surgical intervention [5,6].

In recent years, minimally invasive surgeries such as laparoscopic and robotic surgery have been increasingly used by urologists worldwide [7]. Depending on the type of surgery, these two approaches are mostly performed with the patient lying in the Trendelenburg position and by applying intra-abdominal pressure throughout the induction of pneumoperitoneum. The latter condition has been recently reported as a risk factor for PO-AKI [8]. Reducing the intra-abdominal pressure and the time in the Trendelenburg position has been adopted to reduce the risk of post-operative complications, especially PO-AKIs [1,9,10,11]. 

The occurrence of PO-AKIs is strongly associated with the risk of mortality, co-occurrence of other post-operative complications, increased length of hospital stay and progressive chronic kidney disease [4]. This and the limited treatment options for PO-AKI require great attention to identify means to prevent it [5].

Protein intake in animal models has been proven to be a protective factor for AKIs due to a direct increase in renal blood flow [12]. Moreover, plasma amino acid levels are able to stabilize renal hemodynamics by afferent artery dilatation, which may increase the glomerular filtration rate by 25 to 60% [13]. In addition, peri-operative supplementation of amino acids in patients undergoing cardiac surgery has been proven to have beneficial effects on AKIs by improving the estimated glomerular filtration rate (eGFR) and urine output [14]. 

The aim of the present study was to evaluate if the perioperative administration of intravenous amino acids may reduce the occurrence of PO-AKIs in patients submitted to major laparoscopic urological surgery. 

## 2. Material and Methods

### 2.1. Study Design

This was a before–after clinical study conducted at the Galliera Hospital of Genoa from January 2022 to May 2023. All patients signed an informed consent form on personal data storage and the local ethics committee approved the study (7/2019 id: 4378, amendment 2). 

Three hundred and thirty-one patients older than 18 years undergoing any major urological, minimally invasive surgery were consecutively studied. The presence of preoperative severe kidney chronic diseases (eGFR < 35 mL/min/1.73 m^2^), regardless of the main cause, was the only exclusion criterion. Patients who developed a PO-AKI due to obstructive uropathy were also excluded.

Standard laparoscopies were performed on 164 patients and robot-assisted surgeries were performed on 166 patients, following the indications of the European Association of Urology guidelines (EAU). All patients were treated following a standardized multidisciplinary Enhanced Recovery After Surgery (ERAS) protocol as previously described [1]. Patients undergoing surgery from January to September 2022 (crystalloid group, n =169) were treated with standard intravenous fluid administration and a group of them had also been included in a previous study [1]. Those undergoing surgery from October 2022 to June 2023 (AA group, n = 162) received infusion of intravenous amino acids. Patients of both groups were treated by the same surgical team.

For each patient, the following anamnestic data were recorded: age, sex, body mass index (BMI), American Society of Anesthesiologists (ASA) score, preoperative serum creatinine and hemoglobin. Furthermore, the following comorbidities were evaluated to identify the presence of risk factors for developing PO-AKI: age > 75 years, female sex, hypertension, hyperlipidemia, liver diseases, peripheral vascular diseases, smoking history, diabetes, anemia and cardiovascular diseases [15]. The cumulative number of risk factors per patient was calculated. The data collected intraoperatively were duration of surgery, estimated blood loss, mean arterial pressure and fluid administration. 

### 2.2. Intraoperative Procedure

At admittance to operating room, after positioning a large caliber peripheral venous access (16 G), all patients in the AA group received continuous infusion of Sintamin 10% at 100 mL/h, before anesthesia induction. The intravenous infusion of Sintamin was continued at the same rate for the entire duration of surgery and was reduced to 50 mL/h after recovery from anesthesia throughout post-operative day 1. Other additional crystalloids were administrated per clinical need depending on the patient’s hemodynamics and intraoperative blood loss, always following the restrictive goal-directed fluid therapy imposed by the ERAS protocol [1]. 

The crystalloid group was managed with the same restrictive goal-directed fluid therapy imposed by the ERAS protocol using mostly Ringer’s lactate as the intravenous crystalloid. Post-operatively, the patients of crystalloid group continued the infusion of Ringer’s lactate at 50 mL/h until the end of post-operative day 1. 

All patients started drinking water 2 h after extubation and were allowed free oral water intake 6 h later. 

The intraoperative abdominal pressure induced by pneumoperitoneum was settled at 10 mmHg and maintained using an AirSeal Intelligent Flow System^®^ (ConMed, Utica, NY, USA) or a LexionSystem^®^ (Lexion Medical, St Paul, MN, USA) throughout the whole surgical procedure in both the laparoscopic and robotic-assisted procedures. 

### 2.3. Outcome Measures

Primary outcomes were the incidence and gravity of PO-AKI, defined according to the Kidney Disease Improving Global Outcomes criteria based on creatinine values and urinary output variations [2].

Secondary outcomes were admittance to the intensive care unit, in-hospital length of stay, 30 days re-admission and any kind of complication. The safety of amino acid administration was evaluated by adverse reaction development or an increase in intra-operative blood loss in partial nephrectomies. A cost-effectiveness assessment was conducted by comparing administration of amino acids with Ringer’s Lactate. 

### 2.4. Statistical Analysis

By assuming an effect size = 0.20 (with arcsine transformations of the square root differences between AKI proportions for two distinct patient groups before/after treatment), alpha = 0.05 and power (1 − Beta) = 0.8, a minimum overall sample size of 196 patients was required.

All the variables are expressed as median and interquartile range (IQR) for continuous variables, or as a percentage (%) for categorical variables. The Shapiro–Wilk test was used to evaluate the normal distribution of continuous variables. The Mann–Whitney U-test or Fisher’s exact test was used to evaluate differences between groups for continuous or categorical variables, respectively. The cumulative probability for lack of PO-AKI was calculated. 

Logistic regression was used to identify independent variables potentially associated with PO-AKI assumed as the dependent variable. 

Statistical significance was assumed at two-tailed *p* < 0.05. Statistical analyses were performed by using SPSS, version 27.0 (SPSS, Chicago, IL, USA), software packages. A *p*-value < 0.05 was considered statistically significant. 

## 3. Results

Patients’ characteristics, type of surgery and preoperative data did not differ significantly between groups, except for sex (*p* = 0.028). There were no differences in the duration of surgery, intraoperative mean arterial pressure or intraoperative blood loss between groups (Table 1). 

The total number of PO-AKIs was significantly higher in the crystalloid than the AA group (34 vs. 17, *p* = 0.022), with a significantly lower incidence of KDIGO 1 and 2 PO-AKI in the AA than the crystalloid group (14 vs. 30 *p* = 0.016), whereas no difference was observed in the incidence of KDIGO 3 class of PO-AKIs (3 vs. 5 *p* = 0.724). Considering the overall population of crystalloid and AA groups, no difference was found in the cumulative number of PO-AKI risk factors (1 (1-2) vs. 1 (1-2) *p* = 0.310). The median number of risk factors was not significantly different between the patients who developed PO-AKI and those who did not (2 (1-2) vs. 1 (1-2) *p* = 0.196) (Table 2).

Within the crystalloid groups, there was no significant difference in the median number of risk factors between patients who developed PO-AKI and those who did not (1 (0-2) vs. 1 (1-2), *p* = 0.995). In contrast, within the AA group, those patients who developed PO-AKI had a significantly higher number of risk factors than those who did not (2 (2-4) vs. 1 (1-2), *p* = 0.031). The best cut-off for the number of risk factors to identify PO-AKI determined by ROC analysis was ≥3 (AUROC: 0.701, *p* = 0.007, CI: 0.553–0.850, accuracy 55%, sensitivity 47%, specificity 83%).

In females, the occurrence of PO-AKI was insignificantly different between treatment groups (4/21 vs. 3/32, *p* = 0.477). Direct logistic regression was used to assess the impact of a number of factors on the likelihood of developing PO-AKI. The model contained five independent variables (intravenous amino acid infusion, sex, ASA, duration of surgery, estimated blood loss and intra-operative fluid administration). The full model containing all predictors was statistically significant, X^2^ = 6.553, *p* = 0.010. Only intravenous amino acid infusion made a unique statistically significant contribution to the model with an OR = 0.480, CI:0.250–0.923, *p* = 0.028 controlling for all other factors in the model (Table 3).

In-hospital length of stay was higher in the crystalloid group than in the AA group (respectively, 58 vs. 34 days, *p* = 0.007 and 4 days (3–5) vs. 4 days (4–6) *p* = 0.034). The cumulative number of days of hospitalization was 923 for the 169 patients in the crystalloid group and 752 for the 162 patients in the AA group with a reduction of 171 days and a net estimated saving in hospital costs of EUR 68,400 in eight months. The amount of savings clearly exceeded the increased cost of administering amino acids instead of crystalloids during hospitalization, as the costs for the whole period of 8 months were estimated to be about EUR 1156 (calculating a cost of Ringer’s lactate of EUR 0.40 and of Sintamin of EUR 2.78 per unit). Furthermore, considering an observed median hospital stay of 4 days, the reduction in the duration of hospitalizations by 171 days may have allowed 42 more patients to undergo oncological surgery over the same time period. 

No differences were found in the 30-day readmission rate, as they were all complications due to surgical reasons (urinary fistula, intra-abdominal, blood-serum collection, urinoma, lymphocele). 

Regarding safety, there were no differences between crystalloid and AA groups in terms of intraoperative bleeding assessed by estimated blood loss (200 mL (95–400) vs. 150 mL (50–300) *p* = 0.087) or off-clamp partial nephrectomies (261 mL (100–380) vs. 234 mL (50–265) *p* = 0.768), and no allergic reactions were reported. 

## 4. Discussion

The main findings of this before–after clinical study are that the perioperative administration of amino acids reduced (1) the incidence of mild-to-moderate PO-AKI and (2) the in-hospital length of stay and its related costs. 

A possible explanation of our results could be the normal hyperemic response of the kidneys to a protein workload to enhance the elimination of nitrogenous wastes. Indeed, eating a high protein meal is known to have a great influence on renal perfusion by causing vasodilatation of afferent arteries and a reduction in vascular resistances through local receptors (glutamate receptor N-methyl-D-aspartate) and humoral factors (nitric oxide, insulin, glucagon, prostaglandins, etc.), finally leading to an increase in renal blood flow and the glomerular filtration rate (eGFR) by up to 35% from the baseline [2,13,16].

In agreement with these assumptions, studies on animal models showed that the resulting increase in renal blood flow after a protein meal can protect the kidneys against acute ischemic insults [7,8]. Likewise, a randomized controlled trial in 22 adult cardiac surgery patients demonstrated that amino acid infusion started immediately after surgery can significantly increase renal blood flow and the GFR and optimize renal oxygen consumption in this population [17].

In the present study, a significantly lower number of PO-AKIs was detected in the AA than the crystalloid group thanks to a reduced occurrence of mild-to-moderate AKIs in class KDIGO 1 and 2. 

We interpreted this result by considering that some of our patients possibly had pre-existing subclinical renal damage at hospital admittance with a reduced renal functional reserve, despite the absence of any rise in the serum baseline creatinine level [18]. Nevertheless, this condition may lead to a high susceptibility of the kidneys to new insults and PO-AKI. 

The perioperative increase in renal blood flow and consequently the eGFR via amino acid infusion is only possible in kidneys with a sufficient residual recruitable nephron mass. In fact, patients with less than three risk factors seem to benefit more from perioperative amino acid infusion. Therefore, in the case of severe pre-existing structural nephropathy, amino acid infusion may not be able to reduce the incidence of PO-AKI. This result is supported by the reduction in the incidence of KDIGO 1 and 2 but not KDIGO 3 in the AA group.

There was a statistically significant sex difference between crystalloid and AA groups. Recent animal studies focused on sex differences and AKI, although with some conflicting data, and described an increased susceptibility to AKI development in males due to the presence of testosterone which increases endoplasmic reticulum stress [18,19].

A reduction in PO-AKI leads to a faster enhanced recovery after surgery and a reduced in-hospital length of stay, which may be translated into a global economic saving and the possibility of increasing the monthly number of surgeries. 

Finally, it should be emphasized how amino acid infusion increases renal perfusion without increasing intraoperative blood loss even in partial off-clamp nephrectomies. 

The present study has limitations. First, it was a single-center, before–after design without randomization. Second, consecutive patients underwent major urologic minimally invasive surgery without considering the impact of different types of surgery on renal function. Nevertheless, the absolute and relative numbers of surgery types were similar between groups with the exception of radical prostatectomy. Third, the low occurrence of PO-AKI KIDGO 3 might have affected the results. Fourth, this study was not designed to investigate the underlying mechanisms of intravenous amino acid infusion efficacy and basic experiments, and larger clinical studies are needed to support our results. Fifth, the lack of determination of fractional sodium excretion and urine sediments might have obscured chronic kidney conditions. Sixth, the effects of medications affecting the renin–angiotensin system were not investigated. 

## 5. Conclusions

The results of the present study suggest that perioperative intravenous amino acid infusion in major minimally invasive urological surgery is safe and might be helpful in reducing the incidence of PO-AKI, the hospital length of stay and related hospital costs, thus potentially shortening pre-operative waiting lists. Further randomized trials focusing on selected surgical interventions are needed to better define the efficacy and safety of perioperative intravenous amino acid infusion for the prevention of PO-AKIs and other complications.

## Figures and Tables

**Table 1:** Baseline characteristics of patients and pre-operative and intra-operative data.

	Crystalloid Group (n. 169)	AA Group (n. 162)	*p* Value
Sex, m/f, n	148/21	127/35	0.028
Age, yr	68 (61–73)	68 (61–73)	0.944
BMI, kg/m^2^	25 (23–28)	26 (23–29)	0.378
Comorbidities, n			
Hypertension	88 (52%)	96 (18%)	0.184
Hyperlipidemia	25 (15%)	36 (22%)	0.089
Liver disease	2 (1%)	2 (1%)	0.999
Peripheral vascular disease	6 (4%)	6 (4%)	0.999
Smoking history	26 (15%)	29 (18%)	0.558
Diabetes	15 (9%)	15 (9%)	0.999
Anemia	2 (1%)	3 (2%)	0.679
Cardiovascular disease	24 (14%)	20 (12%)	0.746
Number of PO-AKI risk factors	1 (1–2)	1 (1–2)	0.310
ASA physical status, class	2 (2–2)	2 (2–2)	0.054
eGFR mL/min/1.73 m^2^	84 (66–101)	82 (69–99)	0.974
Creatinine, mg·dL^−1^	0.9 (0.8–1.1)	0.9 (0.8–1)	0.989
Hemoglobin, g/dL	15 (14–16)	14 (13–16)	0.402
Type of surgery, n (%)			
Partial nephrectomy	28 (17%)	29 (18%)	
Radical prostatectomy	102 (60%)	77 (48%)	
Radical nephrectomy	24 (14%)	22 (14%)	
Adrenalectomy	3 (2%)	8 (5%)	
Radical cystectomy	1 (1%)	7 (4%)	
Other	11 (7%)	19 (12%)	
Duration of surgery, min	139 (100–180)	150 (115–201)	0.080
Blood loss, mL	200 (95–400)	150 (50–300)	0.087
Mean arterial pressure, mmHg	79 (73–85)	79 (70–88)	0.966
Intra-operative fluids, L	1.30 (1–1.7)	1.30 (1–1.62)	0.653

BMI, body mass index; ASA, American Society of Anaesthesiology; eGFR, estimated glomerular filtration rate. Data are medians with interquartile range (IQR) or absolute numbers with percentage (%).

**Table 2:** Outcomes and post-operative data.

	Crystalloid Group (n. 169)	AA Group (n. 162)	*p* Value
Clavien–Dindo class, n (%)			
0	114 (67%)	132 (81%)	
1	30 (18%)	20 (12%)	
2	19 (11%)	9 (6%)	
3 (A-B)	4 (2%)	0 (0%)	
4	1 (1%)	0 (0 %)	
5	1 (1%)	1 (1%)	
Acute kidney injury, n (%)	34 (20%)	17 (11%)	0.022
KDIGO grades, n (%)			
1	18 (11%)	10 (6%)	0.169
2	12 (7%)	4 (2%)	0.071
3	5 (3%)	3 (2%)	0.724
Hemoglobin at discharge, g/dL	12 (11–13)	13 (11–14)	0.151
Peak creatinine, mg·dL^−1^	1 (0.9–1.3)	1.1 (0.9–1.3)	0.945
eGFR mL/min/1.73 m^2^ at discharge	80 (63–95)	77 (63–95)	0.980
Creatinine at discharge, mg·dL^−1^	0.9 (0.8–1.1)	1 (0.8–1.2)	0.674
Hospital length of stay, days	4 (4–6)	4 (3–5)	0.034
Days of hospitalization (min–max)	923 (2–49)	752 (2–20)	0.034
Re-admission at 30 days	6 (4%)	13 (8%)	0.100
Clavien–Dindo class, n (%)			
0	161 (95%)	146 (90%)	
1	0 (0%)	1 (1%)	
2	1 (1%)	5 (3%)	
3 (A-B)	7 (4%)	10 (6%)	
4	0 (0%)	0 (0%)	
5	0 (0%)	0 (0%)	
Increase in costs (EUR)	-	1156	-
Saved costs (EUR)	-	68,400	-

KDIGO, Kidney Disease: Improving Global Outcomes; eGFR, estimated glomerular filtration rate. Data are medians with interquartile range (IQR) or absolute numbers with percentage (%), unless otherwise specified.

**Table 3:** Multiple logistic regression analysis of predictors of PO-AKI.

Variables	B	OR	95% CI		*p*
Sex, m/f, n	−0.259	0.771	0.312	1.906	0.574
ASA physical status, class	0.416	1.516	0.834	2.757	0.173
Duration of surgery, min	−0.006	0.994	0.988	1.000	0.064
Blood loss, mL	0.001	1.001	1.000	1.002	0.226
Intra-operative fluids, L	0.000	1.000	0.999	1.001	0.817
Intravenous amino acid infusion	−0.734	0.480	0.250	0.923	0.028

## Data Availability

Data were not inserted in publicly archived datasets but are available anonymized for research purposes upon request to the corresponding author.
